# Analysis of vaginal microbiota before and after treatment of high-grade squamous intraepithelial lesions of the uterine cervix

**DOI:** 10.61622/rbgo/2024rbgo86

**Published:** 2024-12-04

**Authors:** Patrícia Mendonça Ventura, Isabel Cristina Chulvis do Val Guimarães, Luis Guillermo Coca Velarde, Susana Cristina Aidé Viviani Fialho, Douglas Guedes Ferreira, Matheus Madureira Fernandes, Rafael Augusto Chaves Machado

**Affiliations:** 1 Universidade Federal Fluminense Niterói RJ Brazil Universidade Federal Fluminense, Niterói, RJ, Brazil.

**Keywords:** Uterine cervical neoplasms, Cervixuteri, Papillomavirus infections, Microbiota, Vaginosis, bacterial, Squamous intraepithelial lesions of the cervix

## Abstract

**Objective:**

HPV infection is considered the most common sexually transmitted virus today. The persistence of HPV is the main cause for the development of precursor lesions and cervical cancer. There are environmental and non-environmental factors that contribute to the persistence of the virus. Studies indicate a possible relationship between the vaginal microbiota (environmental factor) and the risk of high-grade cervical squamous intraepithelial lesions and cervical cancer. This study evaluates the association between the type of vaginal microbiota and the occurrence of high-grade squamous intraepithelial lesions of the cervix.

**Methods:**

Observational, longitudinal, prospective, and analytical studies carried out between 2019 and 2021, which evaluated the vaginal microbiota of patients diagnosed with high-grade cervical squamous intraepithelial lesion before and after treatment in two collections with an interval of 6 months, using scrapings and vaginal swabs.

**Results:**

In Group I (with lesions) 28 women participated and 29 in Group II (without lesions). According to Nugent, in the initial collection of Group I, 16 women (57%) had lactobacillary microbiota, eight (28%) intermediate, and four (14%) coccus. In Group II, twenty-one (75%) were lactobacillary, one (3%) was intermediate, and seven (24%) werecoccus. With p=0.03.

**Conclusion:**

According to Nugent's criteria, there was an association between the type of vaginal microbiota and the occurrence of high-grade cervical squamous intraepithelial lesions of the cervix. The same was not observed in the Donders classification. Studies with a larger sample are needed to confirm our results.

## Introduction

HPV infection and the persistence of oncogenic subtypes plays a fundamental role in the development of cervical cancer. It is accepted that other causes or cofactors interfere with the development of this condition in women affected by HPV. Recently, several studies have questioned the role of the vaginal microbiota in the process of acquisition and persistence of HPV and the risk of developing cervical cancer.^(1–5)^

The pathogenesis of the association of dysbiosis and high-grade squamous intraepithelial neoplasiaofthecervixis not known. It has been suggested that dysbiosis causes damage to squamous cells, which facilitates the entry of HPV into cervical basal epithelial cells, creating an environment that favors viral replication, leading to the persistence of the lesion and dysplasia. Levels of proinflammatory cytokines are higher in women with dysbiosis, and chronic inflammation is a known factor in the carcinogenesis of numerous tissues in the body.^(5)^ It is known that the main imbalancein the vaginal microbiota is bacterial vaginosis.

The vaginal microbiota is made up of different bacteria that are constantly changing according to the woman's stage of life. During menacme, estrogen plays an important role in main taining an acidic environment in the vagina necessary for a healthy microbiota. During the climacteric period, there is a reduction in estrogen levels, and therefore a reduction in Lactobacillus, favoring an alkaline vaginal environment, which reduces the viability of the healthy endogenous vaginal microbiota and favors the development of GRAM-negative fecal pathogens and other species of bacteria.^(6)^ More than 70% of the human vaginal microbiota is made up of Lactobacillus species, which makes the pH of the vagina acidic (3.8-4.5). Vaginal glycogen and its decomposition products are an important source of energy for Lactobacillus, there for ehigh levels of glycogen are favorable to their growth and proliferation.^(6)^

Recurrent vulvovaginal infections can result in infertility, preterm birth, miscarriage and other infectious diseases. Due to these effects on reproductive health and well-beingin the area of women's health, vaginal infections have become a public health problem world wide.^(7)^

The vaginal microbiota can be classified using the Nugent^(8)^ and Donders^(9)^ criteria. The Nugent method using GRAM-stained smears is the most widely-used microscopic classification of the vaginal microbiota in research environments,^(8–10)^and is considered the most established classification. In recent years, the relationship between the microbiota present in various systems ofthe body and the development of pathologies has been studied.

According to Donders^(9)^ if there are varying sizes of lactobacilli (90% lactobacilli and 10% other bacteria), the microbiota is considered normal, called lactobacillus grade I. Lactobacillus grade II is considered intermediate, composed of 50% lactobacilli and 50 % of other types ofbacteria. If lactobacilli arein greater numbers than other bacteria, it is classified as IIa. If the other bacteria are in greater numbers, it is classified as IIb. Lactobacillus grade III is a complete break with the normal microbiota, with 10%of the morphotypes corresponding to lactobacilli and 90% to other bacteria.^(8)^GRAM staining has greater sensitivity for diagnosing bacterial vaginosis than wet microscopy. Nugent's diagnostic criteria use GRAM staining and are based on scores in which lactobacillus morphotypes receive as core from 1 to 4 according to their predominance, *Gardnerella sp* morphotypes also receive as core from 1 to 4, and *Mobiluncus sp* from 1 to 2. A Nugent score≥7 diagnoses bacterial vaginosis, as core≤3 determines normal microbiota, and a score between 4 and 6 determines intermediate microbiota, which has a controversial interpretation.^(9)^

According to the literature, the main factor that causes the imbalance of the vaginal microbiota is bacterial vaginosis. Suehiroet al^(2)^ analyzed the co-infection rate between HPV and bacterial vaginosis in cervical samples from 278 women through cervical smears, oncotic colpocytology and HPV deoxyribonucleic acid (DNA) analysis. This study concluded that co-infection between bacterial vaginosis and high-risk HPV-DNA was associated with an increased risk of low-and high-grade squamous intraepithelial lesions of the cervix and cervical cancer.

In this study, we sought to analyze the association of the vaginal microbiota with high-grade squamous intraepithelial lesions of the cervix. To date, no publication was found in our search that relates the vaginal microbiota using the Nugent or Donders classification with high- grade squamous intraepithelial lesions of the uterine cervix.

## Methods

This is ananalytical, observational, longitudinal and prospective study that was carried out at the Hospital Universitário Antônio Pedro (HUAP), in Niterói- Rio de Janeiro. Data were collected from March 2019 to September 2021. A convenience sample was used, comprised of women treated at the Colposcopy Outpatient Clinic and the General Gynecology Outpatient Clinic at HUAP.

The inclusion criteria for Group I were women with a histopathological diagnosis of high-grade cervical squamous intraepithelial lesion (CIN II and III), aged 25 to 64 years. In Group II, women with negative oncotic cytology were included, aged between 25 and 64 years. The exclusion criteria for the study include: pregnant women, women using an intrauterine device or vaginal ring, and women with cancer.

The non-exclusion of immunosuppressed patients was due to the fact that we were studying the microbiota of women that already have high-grade lesions, no matter if immunosuppressed. The population sample was for convenience and we would like to observe the characteristics of the microbiota in all women with high-grade squamous intraepithelial lesions.

The presence of Group II in the study allowed the comparison ofthe characteristics of the vaginal microbiota in patients with negative cytology and patients diagnosed with high-grade cervical squamous intraepithelial lesion. Group II was composed of women reated at the general gynecology outpatient clinicoh HUAP, with negative cytology.

**Figure 1:**
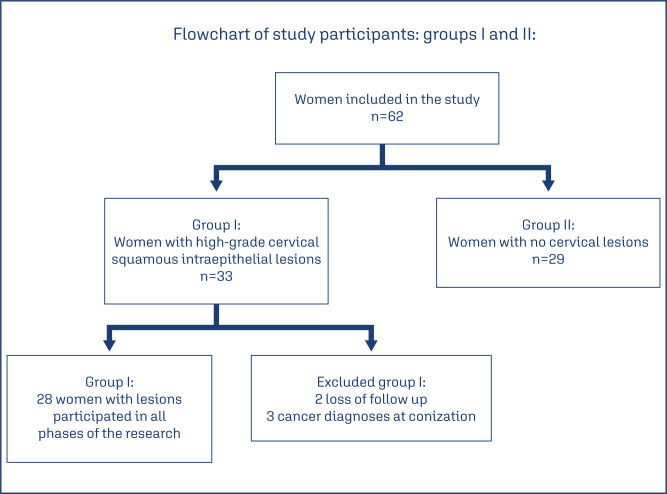
Study participants: Groups I and II

There were initially 62 women included in the study, 33 women from Group I, and 29 women from Group II. Initially, alarger sample was planned, but due to the coronavirus disease, surgical and out patient services at HUAP were restricted, with a consequent reduction in the population served at the hospital. Of the 33 women with lesions, 3 of them were excluded due to a diagnosis of cancer during surgical treatment. There were 30 women remaining in Group I, of which two were lost to follow-up during reevaluation after 6 months, leaving 28 women in Group I who participated in all phases of the study (Figure 1).

The following variables were analyzed in both groups: smoking, multiparity, immunosuppression and use of oral hormonal contraception through a questionnaire administered when the patient was admitted to the outpatient clinic. The vaginal microbiota was classified according to Donders and Nugent criteria. For this, two vaginal swabs were collected: one for Candida culture, and the other for aerobic bacteria culture, collected before the surgical procedure and 6 months after treatment, in Group I. A scraping from the vaginal wall was also collected to analyze the type of vaginal microbiota using a microscope with an ocular lens with a magnification of 10 and an objective lens with a magnification of 40 and were associated with staining using the Gram method with the aim of evaluating the proportion of leukocytes and epithelial cells, percentage of toxic leukocytes, percentage of basal/parabasal cells, the amount of Lactobacillus, and the identification of pathogens. The sample was collected before the surgical procedure and 6 months after treatment, in Group I. For initial analysis of the type of vaginal microbiota, we performed the same procedures described above in women without Group II in two moments with an interval of 6 months.

For statistical analysis, programs were used that differentiate between categorical and numerical variables. Numerical variables (age) and categorical variables (smoking, oral hormonal contraceptive, multiparity, immunosuppression, vaginal microbiota according to Nugent and Donders, *Candida sp*., aerobic bacteria) were evaluated. Groups I and II were compared. To analyze the numerical variable, the Wilcoxon Rank Sum Test With Continuity Correction (MANN-WHITNEY) test was used. When the p-value < 0.05 there is a difference between the groups analyzed.

For the analysis of categorical variables, the Fisher'sExactTest for Count Data and the Test based on normal distribution for paired proportions were used. When the p-values <0.05, there is a difference between the groups analyzed.

For statistical analysis, data were compiled in a Microsoft Excel 2019 spread sheet and analyzed using the free program R version 3.6.1.

The present study was approved by the Research Ethics Committee of the Faculty of Medicine of HUAP under opinion (5.147.613) number of certificate of presentation for ethical assessment (CAAE) 17565519.9000.0.5243.

## Results

In Group I, the mean age of the women was 43 years and the median was 46. In Group II, the mean age was 45 years and the median was 47. The analysis of the age variable is similar in both Groups (p = 0.64) (Table 1).

**Table 1:** Characteristics of the study population of Groups I and II

Characteristics	Group I	Group II	p-value
	n=28	n=29	
	n(%)	n(%)	
Meanage	43	45	0.64
Median	46	47	
Smokers	6(21)	1(3)	0.05
Hormonalcontraception	8(28)	8(27)	1
Multiparty	11(39)	13(44)	0.79
Immunosupression	4(14)	0(0)	0.05

Regarding immunosuppression, in Group I, four women (14%) were immunosuppressed, while no women in Group II were immunosuppressed (p=0.05). Therefore, there is evidence that there is no significant difference between the percentages of immunosuppressed people between the two groups. The causes of immunosuppression in Group I were: pemphigus, kidney transplantation, systemic lupus erythematosus and autoimmune enteropathy. Five women in Group I (17%)and four in Group II (13%) had *Candida sp* in the first collection. Therefore, the groups were similar in relation to the presence of Candida sp (p = 0.72) (Table 2). Immunosuppressed patients were only present in Group I, there were no immunosuppressed patients in Group II. We chose to keep immunosuppressed patients since our population sample was for convenience and we would like to observe the characteristics of the microbiota in all women with high-grade squamous intraepithelial lesions.

**Table 2:** Characteristics of the vaginal microbiota at baseline and 6 months, correlating both groups at each of the study moments

Characteristics		Group I	Group I	Group II	Group II	p-value	p-value
		Baseline	After 6 months	Baseline	After 6 months	Baseline	After 6 months
		n=28	n=28	n=29	n=29		
		n(%)	n(%)	n(%)	n(%)		
*Cândidasp.*		5(17)	5(17)	4(13)	3(10)	0.72	0.47
Vaginal microbiota						0.48	0.31
Donders							
	Type I	5(18)	9(32)	9(31)	13(45)		
	Type IIa	12(43)	15(54)	12(42)	10(34)		
	Type IIb	9(32)	4(14)	5(17)	4(14)		
	Type III	2(7)	0(0)	3(10)	2(7)		
Microbiota						0.03	0.14
Nugent							
	Lactobacillary	16(57)	21(75)	21(72)	23(79)		
	Intermediate	8(29)	6(21)	1(4)	2(7)		
	Coccus	4(14)	1(4)	7(24)	4(14)		
Aerobicbacteria		5(17)	4(14)	0(0)	1(3)	0.02	0.19

Six months after the intervention,*Candida sp* was found to be present in five women (17%) in Group I. In Group II, three women (10%) had *Candida sp* in the second collection after 6 months. The groups remained homogeneou seven after the intervention (p=0.47). Regarding the analysis of the microbiota using the Donders classification, in the evaluation before treatment (Group I), five women (17%)had their microbiota classified as type I, fourteen (42%) as type IIa, nine (32%) as type IIb, and two(7%) as type III. In Group II, in the initial collection, nine women (31%) corresponded to type I microbiota, twelve (41%) to type IIa, five(17%) to IIb, and three (10%)to type III. The distribution of the type of microbiota between Groups I and II in the initial analysis was similar (p=0.48).

This same assessment was carried out in Group I 6 months after the intervention and in Group II 6 months after the first collection. In Group I, nine (31%)women were found with type I microbiota, fifteen (53%) with type IIa, four (14%) with type IIb, and none (0%) with type III. In Group II, a sample collected 6 months after the first collection identified thirteen (44%) women with type I microbiota, ten (34%)with type IIa, four (13%) as type IIb, and two (6%) as type III. This analysis also showed no significant difference between the groups (p=0.31). In the analysis of the vaginal microbiota according to the Nugent classification, in the initial collection of Group I, sixteen women (57%) had lactobacillary microbiota, eight (28%) intermediate, and four (14%) cocceous. In Group II, 21 women (75%) had lactobacillary, one (3%) intermediate, and seven (24%) coccyx. With p = 0.03. Therefore, it can be stated that there is a difference between the distribution of the microbiota between Groups I and II before the intervention according to the microbiota classification by Nugent.

In the evaluation of the microbiota according to Nugent in the second collection of group I, 21 women (75%) had lactobacillary microbiota, six (21%) had their microbiota classified as intermediate and one (3%) had their microbiota classified as coccus. In Group II, 23 women (79%) had their microbiota classified as lactobacillary, two (6%) intermediate, and four (13%) as coccus microbiota. Therefore, we did not observe a statistically significant difference between the samples evaluated in relation to the analysis of the vaginal microbiota 6 months after treatment and in the collection 6 months after the first in the control group (p=0.14)

Regarding the growth of aerobic bacteria, before treatment, the presence of aerobic bacteria in group I was observed in five women (17%) and in Group II, no woman showed growth of aerobic bacteria (p=0.02). Therefore, there is an association regarding the presence of pre-treatment aerobic bacteria in both groups. There was a greater presence of aerobic bacteria in Group I. In the evaluation 6 months later, in Group I, growth of aerobic bacteria was identified in four women (14%), and in Group II, in only one woman (3%). There was no statistically significant difference between the groups analyzed (p= 0.19). The aerobic bacteria identified before the intervention were *Staphylococcus aureus* (intwopatients), *Enterococcus faecalis* (in two patients), *Klebsiela pneumoniae* (in one patient). And after treatment: *Streptococcus agalactae* (two patients), *Klebsiela pneumoniae* (one patient)and *Escherichia coli* (one patient). There was no growth of aerobic bacteria in the culture of Group II patients during the initial collection, and only one woman was found to have *Escherichia coli* 6 months later.

Table 3 describes the distribution of the type of vaginal microbiota (normal and altered) according to Donders and Nugent at baseline and after 6 months in Groups I and II. We consider type I and IIa microbiota as normal microbiota in the Donders classification, and type IIb and III as altered microbiota. In Nugent, the microbiota considered normal corresponds to the lactobacillary microbiota and the microbiota composed of the intermediate and coccus types is altered.

**Table 3:** Characteristics of the vaginal microbiota at baseline and 6 months-analyzing the association of the vaginal microbiota between the same group at both moments of the study

Characteristics		Group I	Group I	Group II	Group II	p-value	p-value
		Baseline	After 6 months	Baseline	After 6 months	Group I	Group II
		n=28	n=28	n=29	n=29		
		n(%)	n(%)	n(%)	n(%)		
Microbiota Donders						0.00	0.15
	Type A	17(61)	24(86)	21(73)	23(79)		
	TypeB	11(39)	4(14)	8(27)	6(21)		
Microbiota Nugent						0.06	0.15
	Type A	16(57)	21(75)	21(72)	23(79)		
	TypeB	12(43)	7(25)	8(28)	6(21)		

Microbiota Donders: Type A (normal) = microbiota type I + IIa; Type B (altered) = microbiota type IIb+ III Nugent: Type A (normal): lactobacillary microbiota; Type B (altered): intermediate+ coccus microbiota

The presence of risk factors smoking, hormonal contraception, immunosuppression and multiparity were not associated with the type of vaginal microbiota in Group I in the first sample collected in the study (time 0), as the p-value of these analyzes was ≥ 0.05 (Table 4).

**Table 4:** Analysis of risk factors and normal and altered vaginal microbiota according to the Donders and Nugent Classification in women with high-grade squamous intraepithelial lesions of the uterine cervix

Group I: baseline	Smoker n(%)	Hormonal Contraception n(%)	Immunosuppression n(%)	Multiparty n(%)
**Microbiota**				
**Donders**				
Type A	3 (10)	5(18)	4 (14)	8(28)
Type B	3 (10)	3(10)	0(0)	3(10)
p-value	0.65	1.00	0.13	0.43
**Microbiota**				
**Nugent**				
Type A	5 (18)	7 (25)	4 (14)	9(32)
Type B	1 (3)	1(3)	0 (0)	2(7)
p-value	1.00	1.00	0.61	0.70

## Discussion

The study that is most similar to ours was that of Wiik et al^(11)^ This is a Norwegian study carried out between 2005 and 2007 that analyzed the cervical microbiota in women with high-grade cervical intraepithelial neoplasia before and after electro surgery. They concluded that there was a reduction in the group of non-*Lactobacillus* bacteria in patients undergoing surgery evaluated at 6and 12 months in comparison to before treatment, and a tendency to see an increase in *Lactobacillus*. The results were not significant for individual bacterial species, likely due to small sample numbers.

Zhang et al^(12)^ evaluated the cervical microbiota before and 3 months after loop electrosurgical excision. This study included 26 women with high-grade cervical intraepithelial neoplasia of the cervix. Cervical swabs were collected and the microbiota was analyzed by sequencing the 16S ribosomal RNA gene. A decrease in cervical microbial diversity was observed after 3 months of treatment. A significant shift of the community type of *Prevotella* and the lack of a dominant species for the *Lactobacillus iners* dominated microbiome were observed after surgery. In particular, *Leptotrichia* and *Clostridium* decreased even more after treatment. The results suggested that 3 months after surgery, the cervical microbiome showed a reduction in microbiota diversity, with a predominance of Lactobacillus.^(12)^

The design of this study does not allow inferring cause and effect, that is, the design only allows us to say whether women with high-grade squamous intraepithelial lesions more frequently presented abnormal flora than women without high-grade squamous intraepithelial lesions. No association was identified between the type of vaginal microbiota and the occurrence of the lesions of the cervix according to the Donders classification (p=0.48). However, according to Nugent, the statistical analysis showed significance (p=0.03), demonstrating that there is an association between the type of vaginal microbiota and the occurrence of lesion using the most established classification in microbiota analysis.

In the present study, 43% of women with high-grade squamous intraepithelial lesions of the uterine cervix had their microbiota classified as dysbiotic (intermediate or coccus microbiota) according to Nugent, while 57% had a normal microbiota (lactobacillary). According to Donders, 61% had normal microbiota, and 39% had dysbiotic microbiota. In the group of women with normal oncotic cytology, 75% of patients had a lactobacillary microbiota, and 73% had a normal microbiota by Donders. Therefore, in our studied sample, a predominance of normal vaginal microbiota was evident in patients without high-grade lesions. After treatment, an increase in the number of normal vaginal microbiota (86%) and lactobacillary microbiota (79%) was observed in the group with lesions. This change in the microbiota pattern showed statistical significance according to the Donders Classification analysis. Therefore, the treatment appears to have an influence on the type of microbiota based on this classification.

Regarding bacterial diversity in the vaginal microbiota, in our study, greater growth of aerobic bacteria was observed in women with high-grade cervical squamous intraepithelial lesions before the intervention (17%), while in Group II, no woman presented aerobic bacteria. This analysis showed that there was some association regarding the presence of aerobic bacteria before the intervention in both groups. This is compatible with the literature that states that the healthy microbiota is predominantly composed of lactobacilli and the growth of aerobic bacteria demonstrates a change in this balance. After treatment, no statistical significance was found in this comparison.

Regarding the association of risk factors with the type of vaginal microbiota, according to the large IARC study, women with seven or more pregnancies have a 4 times greater risk of developing cervical cancer or high-grade squamous intraepithelial lesions of the cervix compared to nulliparous women.^(13)^ In our study, there was no evidence of an association between multiparity and the type of vaginal microbiota using both the Nugent and Donders classification.

The use of synthetic hormones for contraceptive purposes, both combined and isolated progestogens, impact the vaginal microbiota, according to Mitra et al^(3)^. Mitra states that these medications reduce recurrence (31%), prevalence (32%) and the incidence (18%) of bacterial vaginosis. In our study, there was no association between the use of hormonal contraceptives and the type of vaginal microbiota in the two classifications used.

Immunosuppression is a known risk factor for HPV infection and its persistence. In immunosuppressed patients, HPV is more common, the lesions are more exuberant and recur more frequently, they are more difficult to treat, and they are generally lesions caused by oncogenic HPV, which are more predisposed to the emergence of cancer. Women living with HIV/AIDS are five times more likely to develop cervical cancer compared to the general population.^(13)^ In our study, in Group I there were four immunosuppressed patients. However, no relationship was observed between immunosuppression and the type of vaginal microbiota.

Mitra et al^(3)^also highlights that smoking is a factor that influences the composition of the vaginal microbiota, being related to the increase in vaginal microbial diversity and the reduction of *L.crispatus,* which is in agreement with Ventolini et al^(14)^ who stated that the vaginal microbiome dominated by *L*. *iners* was associated with a greater chance of HPV infection and progression to cervical neoplasia compared to the microbiota dominated by *Lcrispatus.* In our study, there was no association between smoking and the type of vaginal microbiota according to the Nugent and Donders classification.

*Candida albicans* is the most common fungus in the human microbiome. It has been identified in the vaginas of approximately 20% to 30% of asymptomatic women with culture methods and in 65% with molecular methods. When symptomatic, it is referred to as vulvovaginal candidíase is and is caused by *C. albicans* in 80% to 92% of cases. Recently, the hypothesis was raised that *C. albicans* has potential benefits for the health of the host by mediating the inhibition of the migration of *Escherichia coli* from the rectum to the bladder, protecting against urinary tract infection, and its role in the development of the immune response of the human intestinal mucosa. Lactobacilli in the vagina are responsible for inhibiting *Candida* from becoming pathogenic through the production of lactic acid, bacteriocins, hydrogen peroxide and biosurfactants. Vaginal bacteriado not prevent *Candida* colonization, but they do prevent its proliferation.^(15)^ In our study in Group I, 17% of patients had *Candida sp* both before and after the intervention and in Group II, 13% had it before and 10% after 6 months. No difference was observed in the presence of *Candida sp* before and after treatment of high-grade squamous intraepithelial lesion in Groups I and II. Therefore, the presence of *Candida* in both groups did not appear to be influenced by the treatment.

The strong point of this study is that it is unprecedented in evaluating the association of vaginal microbiota using the Nugent and Donders classification with high-grade squamous intraepithelial lesions of the uterine cervix.

The small sample size was a limitation in this study. Initially, a larger sample was planned, but due to the COVID-19 pandemic, surgical and outpatient services at HUAP were restricted, with a consequent reduction in the population served at the hospital.

## Conclusion

Using the classification of the vaginal microbiota according to the Nugent criteria, it was determined that there is an association between the type of vaginal microbiota and the occurrence of high-grade cervical squamous intraepithelial lesions of the cervix. Women diagnosed with high-grade cervical squamous intraepithelial lesions presented a high frequency of abnormal vaginal microbiota. The same was not observed by the Donders classification, whose analysis did not show statistical significance. The association between cervical cancer risk factors and the type of microbiota has not been verified. This analysis did not show statistical significance for any risk factor. Our results showed a predominance of normal vaginal microbiota in group II patients at baseline and after 6 months. Group I showed a percentage increase in the number of women with normal vaginal microbiota by both Donders and Nugent after treatment. This change in the microbiota pattern showed statistical significance according to the Donders Classification analysis. Therefore, the treatment can modify the vaginal microbiota, favoring a reduction in the altered vaginal microbiota. The treatment appears to reduce the presence of aerobic bacteria in the vaginal microbiota. In both Group I and Group II, no difference was observed in the presence of *Candida sp* before and after surgical treatment of high-grade squamous intraepithelial lesions. More studies are needed to clarify the influence of the vaginal microbiota on cervical carcinogenesis.
